# Factors related to substance use treatment attendance after peer recovery coach intervention in the emergency department

**DOI:** 10.1016/j.dadr.2022.100093

**Published:** 2022-09-10

**Authors:** Orrin D. Ware, Megan E. Buresh, Nathan A. Irvin, Maxine L. Stitzer, Mary M. Sweeney

**Affiliations:** a School of Social Work, The University of North Carolina at Chapel Hill, 325 Pittsboro Street, Chapel Hill, NC 27599, USA; b Johns Hopkins University School of Medicine; c Department of Medicine, Division of Addiction Medicine, 5200 Mason F. Lord Drive, Baltimore, MD 21224, USA; d Department of Epidemiology, Johns Hopkins Bloomberg School of Public Health, 615 N. Wolfe Street, Baltimore, MD 21205, USA; e Department of Emergency Medicine, 4940 Eastern Avenue, Baltimore, MD 21224, USA; f Department of Psychiatry and Behavioral Sciences, Behavioral Pharmacology Research Unit, 5510 Nathan Shock Drive, Baltimore, MD 21224, USA; g Friends Research Institute, 1040 Park Avenue, Baltimore, MD 21201, USA

**Keywords:** Emergency departments, Screening and brief intervention, Referral and consultation, Peer recovery coach, Substance use disorder, Insurance

## Abstract

**Introduction:**

Brief intervention with peer recovery coach support has been used to generate referrals to substance use disorder treatment from the emergency department (ED). This retrospective study evaluated factors associated with successful linkage to treatment following brief intervention in the ED.

**Methods:**

Data were extracted from the electronic health record for patients who were referred to substance use treatment from the ED and for whom follow-up data regarding treatment attendance was available (*n*=666). We examined associations between demographic and insurance variables, substance use, mental health diagnosis, prior abstinence, and stage of change with successful linkage to substance use treatment after ED referral.

**Results:**

The sample was majority male (68%), White (62%), and had a mean age of 43 years (SD=12). Medicaid was the most common insurance (49%) followed by employer/private (34%). Multivariable logistic regression determined patients with Medicaid (OR=2.94, 95% CI:2.09-4.13, p=<.001), those who had a documented alcohol use disorder diagnosis (OR=1.59, 95% CI:1.074-2.342, p=.02), and those in the “Action” stage of change (OR=2.33, 95% CI:1.47-3.69, p=<.001) had greater odds of being successfully linked to treatment.

**Conclusions:**

These results identify characteristics of patients available in the health record to determine who is more likely or less likely to attend substance use treatment following ED referral. Given appropriate screening, this information could be used to direct standard care resources to those with high likelihood of treatment attendance and strengthen follow-up interventions with peer recovery coaches for those with lower likelihood of treatment attendance.

## Highlights

• The emergency department (ED) is a prime location for substance use intervention.
• Peer support may facilitate linkage to treatment, but important factors are unknown.
• We used electronic health record data to evaluate patient factors related to linkage.
• Medicaid insurance was positively associated with being linked to treatment.
• The action stage of change was positively associated with treatment linkage.

## 1 Introduction

It is both common and costly for patients with substance use disorders to present to a hospital emergency department (ED). A substantial proportion of ED patients screen positive for high-risk alcohol and illicit substance use. One study of over 14,500 ED patients using standardized measures of alcohol and drug use found 45% of patients reported at-risk alcohol use in the past year, 22% had used drugs in the past 30 days, and 17% had moderate to severe drug problems (Sanjuan et al., 2014). Further, substance-use related diagnoses and overdoses are increasingly prevalent in the ED (Liu et al., 2020; Moore et al., 2017; Vivolo-Kantor et al., 2020; Vivolo-Kantor et al., 2018; White et al., 2018). From 2016 to 2017, ED visits due to opioid overdoses increased nearly 30% in an analysis spanning 45 US states, with an increase of 21% in the Northeast region (Vivolo-Kantor et al., 2018). During the COVID-19 global pandemic, there has been an approximate 10% increase in all substance overdoses relative to before COVID-19 (Holland et al., 2021; Soares et al., 2022). Patients with substance use disorder also show greater ED utilization relative to matched controls (Bahorik et al., 2018). In addition, alcohol, opioid, and stimulant-related ED services cost over 1.7 billion in 2017 (Karaca & Moore, 2020). Thus, EDs are important points of entry and expense to the healthcare system for patients with substance use disorders.

In response, EDs have developed programs to screen patients for alcohol and drug use, provide brief intervention, and initiate referral to substance use treatment based on strategies originally developed for alcohol use disorder (Barata et al., 2017; Hawk & D'Onofrio, 2018; Kaczorowski et al., 2020; Kaner et al., 2018; Landy et al., 2016; Monico et al., 2020; Siegel et al., 2021; Webb et al., 2021). Brief intervention provides awareness about harmful substance use risks with a focus on changing behavior (Substance Abuse and Mental Health Services Administration, 2022). Existing data suggests that brief intervention in the ED setting among people who use alcohol or drugs is feasible (Barbosa et al., 2020; Cowell et al., 2017; Pringle et al., 2018), cost effective (Barbosa et al., 2020; Pringle et al., 2018), successful in referring patients to treatment (Kaczorowski et al., 2020), and can result in reduced substance use (Barata et al., 2017; McCall et al., 2021; Waller et al., 2019), decreased future medical costs (Barata et al., 2017; McCall et al., 2021; Pringle et al., 2018), and decreased ED utilization (Barata et al., 2017). Nevertheless, results are not uniformly positive, with some research demonstrating little long-term reductions in substance use following brief intervention with referral in the ED (Bogenschutz et al., 2014; D'Onofrio et al., 2017; D'Onofrio et al., 2015; Hatch-Maillette et al., 2020; Merchant et al., 2018; Saitz, 2018). One contributing factor may be that some patients ultimately do not attend their treatment despite expressing interest in treatment during the brief intervention. Little is known about the factors that are related to treatment attendance following brief intervention in the ED, but prior data suggests having insurance, older age, and readiness to change may be important (Blow et al., 2010). Racial disparity has also been observed, such that Black patients are less likely to be linked to treatment from the ED relative to White patients (Webb et al., 2021). Similarly, gender disparity has been observed, as women are less likely to be linked to treatment from the ED compared to men (Amaducci et al., 2020). These systematic gender and racial disparities to treatment entry are observed in national data such that there is a greater discrepancy between treatment need and treatment receipt among women relative to men and among Black and Hispanic persons relative to White persons (Martin et al., 2021). If clinicians are aware of risk factors for failure to attend treatment, they might identify those most likely to benefit from treatment referrals, while developing novel strategies to improve success rates for those who are less likely to attend treatment. This retrospective analysis used electronic health record data to compare persons who did and did not attend treatment following a brief peer support intervention and active treatment referral in the ED. The purpose was to identify factors related to successful versus unsuccessful linkage to treatment among substance use patients in the ED.

## 2 Material and methods

### 2.1 Brief intervention and referral to treatment program

The Johns Hopkins Bayview Medical Center (JHBMC) in Baltimore, Maryland utilizes the screening, brief intervention, and referral to treatment (SBIRT) model. A positive screen from the triage nurse, defined by the JHBMC as a score of 7 or higher on the three-item Alcohol Use Disorders Identification Test (AUDIT-C) to prioritize very high risk drinking given patient volume in the ED (i.e., a brief three item screener for at-risk drinking with scores ranging from 0-12; other clinical settings may use a cutoff of 3 for women and 4 for men) (Bradley et al., 2007; Bush et al., 1998) or by any self-reported illicit substance use during the past 12 months according to a standardized form in the electronic health record, triggers an automated response in the patient record prompting contact with a peer recovery coach (also called peer support specialist). These specialists are trained to provide brief intervention with people who use substances. The brief intervention consists of assessment of patient substance use (using standardized metrics already described), assessment of readiness for treatment and other patient needs, providing encouragement for the patient to consider treatment, and initiation of referrals to treatment (Eddie et al., 2019; Kleinman et al., 2021). The peer recovery coaches in this healthcare setting received approximately 57 hours of structured training on brief intervention strategies as part of a collaboration with a local consulting firm (Mosaic Group; http://www.groupmosaic.com/) with expertise in substance use program implementation with a particular focus on brief interventions and are supervised by physician and nursing staff. Other training topics include the role, ethics and professional boundaries of peer recovery coaches, recovery and wellness, substance use treatment modalities, and the stages of change from the Transtheoretical Model, which was selected to help peers evaluate readiness to enter treatment (Prochaska & DiClemente, 1983). The stages of change include “precontemplation” (not considering behavioral change), “contemplation” (considering behavioral change), “preparation” (planning to make actionable behavioral change), “action” (initiating behavioral change), and “maintenance” (continuing behavioral change) (Prochaska & DiClemente, 1983). Peer recovery coaches are also trained to enter data about their encounter into the patient electronic health record, which has standardized responses for each component of the intervention and follow-up.

Data entered into the electronic health record (Epic Systems) by the triage nurse at ED intake and subsequently by peer recovery coaches include demographic characteristics (age, race, ethnicity), the AUDIT-C (Bush et al., 1998), self-reported past 12-month substance use, presence of a mental health diagnosis (e.g., major depression), whether the patient had a prior sustained period of abstinence (based on patient and peer judgment of sustained abstinence), and patient stage of change (Prochaska & DiClemente, 1983). Patient stage of change was assessed according to peer interaction with the patient throughout the brief intervention and was captured when the peer recovery coaches responded to a multiple-choice item in the electronic health record workflow to indicate stage of change. In the electronic health record, the multiple-choice stage of change item was listed as “Patient's stage of change” with the following response options: Precontemplation, Contemplation, Preparation, Action, Maintenance, and Relapse. Peer recovery coaches summarize information about patients collected from the brief intervention based on their training, personal judgment, and available health record information into dichotomous drop down options (Yes/No) for consistent entry into a peer recovery coach-specific form in the electronic health record including: whether patients had a mental health diagnosis (e.g., major depression; bipolar disorder), whether patients had a prior sustained period of abstinence (any length; according to peer judgment), and to document whether patients were subsequently linked to treatment. The mental health diagnosis item was labeled in the electronic health record as “Current Mental Health Diagnosis?” to which the dichotomous response options of Yes and No were present. The prior period of abstinence item was labeled in the electronic health record as “Has the patient ever had sustained abstinence?” with Yes and No as response options. There are also opportunities to record other pertinent information in free text fields. Additional substance use information from the health record included whether a substance use disorder diagnosis was present in the electronic patient problem list. The problem list documents all patient medical concerns for clinical and billing purposes. If a need for treatment is identified based on the brief intervention, the peer recovery coach works with the medical team to make an appointment for an appropriate treatment modality (e.g., intensive outpatient services, residential/inpatient services) according to patient need and preference and treatment availability. The peer recovery coach follows up with the treatment program within 1 week of the ED visit to determine whether the patient attended their intake appointment. The follow-up linked to treatment item was labeled in the electronic health record as “Patient admitted for treatment?” with Yes and No as response options.

### 2.2 Study sample

This study used clinical data entered into the electronic health record as part of usual care; no informed consent was obtained for research. Data were extracted with the assistance of the Johns Hopkins Core for Clinical Research Data Acquisition and were de-identified prior to analysis by the study authors. All study activities were reviewed by the Johns Hopkins Medicine Institutional Review Board and determined to be exempt research. Demographic, insurance, substance use, and brief intervention data were extracted from the electronic health record for all Johns Hopkins Bayview Medical Center ED patients ≥18 years old who received a referral to substance use treatment from July 1, 2017 to July 1, 2019. To avoid duplicate cases, only the first ED visit during this timeframe that resulted in a referral to treatment was included. The initial query included 1841 individual patient records. Patient records were excluded if there was: no past 12-month self-reported substance use according to the structured intake assessment nor any substance use disorder (other than tobacco use) documented in the electronic problem list (*n*=303), no follow-up data for treatment linkage (*n*=702), or internally conflicting data (e.g., both yes and no values) for follow-up treatment linkage (*n*=171). Little's Missing Completely at Random (MCAR) test was conducted on the initial query data (*n=*1841) using IBM SPSS Version 25 including variables planned for the logistic regression (see section 2.3) and provided a value of *p*=.21. Because these findings support MCAR and suggest there is no bias introduced by exclusion, patient records without key variables were excluded as described above. A final sample of 666 individual patients were included in the comparison of persons who were (*n*=383) and were not (*n*=283) linked to treatment.

### 2.3 Data analysis

The present analysis consisted of electronic health record data from standard information collected by clinical or intake staff (e.g., demographic and insurance information), standardized screening/triage data collected in the ED regarding past 12-month substance use (Yes/No for each substance), and intervention data entered by the peer recovery coach (e.g., stage of change, presence of a mental health diagnosis, linkage to treatment). Further, we queried the patients’ electronic health record problem list for the presence of an alcohol or substance use disorder to supplement intake data for 12-month self-reported substance use according to ED assessment.

Multivariable logistic regression was used to examine factors related to whether patients successfully attended/were linked to treatment (Yes/No). Predictors in the model may be found in Table 1 and include age, sex, race, insurance (Medicaid or other insurance types: employer/private, Medicare, or self-pay), stage of change at the time of the brief intervention (action or other stage of change, including pre-contemplation, contemplation, preparation, maintenance or unknown). Other variables including presence of a mental health diagnosis, presence of a prior sustained period of abstinence, presence of an alcohol use disorder diagnosis in the problem list, opioid use, cocaine use, and cannabis use were dichotomized as yes/no.

**Table 1:** Variables as included in the multivariable logistic regression model

Variable	Reference Group	Comparator(s)
Age^1^	N/A (Continuous)	
Sex^1^	Male	Female
Race^1^	White	Black, Other
Medicaid^1^	Medicaid insurance	Other insurance/self-pay
Alcohol use disorder diagnosis	Alcohol use disorder diagnosis^2^	Not present
Cocaine use	Used cocaine in the past 12 months^3^ OR cocaine use disorder diagnosis^2^	Not present
Opioid use	Used opioids in the past 12 months^3^ OR opioid use disorder diagnosis^2^	Not present
Cannabis use	Used cannabis in the past 12 months^3^	Not present
Mental health diagnosis	Recovery coach indicated patient had a mental health diagnosis^4^	Not present
Prior period of abstinence	Recovery coach indicated patient had a prior period of abstinence^4^	Not present
Stage of change	Action stage of change^4^	Other
Linked to treatment	Yes, linked to treatment	Not linked to treatment

1 As indicated by the basic demographic information in the electronic health record

2 As extracted from the general medical problem list in the electronic health record

3 As indicated by the Emergency Department intake assessment via the triage nurse

4 As recorded by the peer recovery coach in the brief intervention documentation in the electronic health record

Medicaid was compared to other insurances because it was the most common insurance. Ethnicity was not included in the analyses due to a low frequency of individuals identifying as Hispanic. For cocaine and opioid use, we used either self-reported past 12-month use according to ED assessment OR a use disorder documented in the problem list for the variable in the model because cocaine and opioid use disorders were not commonly documented (5% or less of the sample). Cannabis use disorder was not documented for any patients so only past 12-month cannabis use was used in the model. Other drug use or use disorders (e.g., methamphetamine) were not included due to low prevalence of documented substance use disorders related to these drugs (≤5%). “Action” was compared to other stages of change because it was thematically related to initiating treatment (Prochaska & DiClemente, 1983).

## 3 Results

### 3.1 Characteristics of sample

Table 2 describes the sample. Most of the sample reported a prior period of sustained abstinence from substances (62%; *n*=412). Peer recovery coaches noted a current mental health diagnosis (Yes/No) in 32% (*n*=217) of patients. Most of the sample were in the “preparation” stage of change (55%; *n*=367).

**Table 2:** Characteristics of patients referred to treatment from the Emergency Department

	Total Sample n=666, (100%)	Linked to Treatment n=383 (57.5%)	Not Linked to Treatment n=283 (42.5%)
Age, mean (SD)	43.1 (12.0)	42.6 (11.6)	43.7 (12.6)
Sex (%)			
Male	453 (68%)	256 (67%)	197 (70%)
Female	213 (32%)	127 (33%)	86 (30%)
Race (%)			
Black or African American	214 (32%)	122 (32%)	92 (33%)
White	414 (62%)	237 (62%)	177 (63%)
Other	38 (5%)	24 (6%)	14 (5%)
Ethnicity (%)			
Hispanic	23 (4%)	15 (4%)	8 (3%)
Not Hispanic	640 (96%)	367 (96%)	273 (97%)
Missing	3 (<1%)	1 (<1%)	2 (<1%)
Insurance (%)			
Medicaid	326 (49%)	233 (61%)	93 (33%)
Employer or Private	228 (34%)	95 (25%)	133 (47%)
Medicare	74 (11%)	39 (10%)	35 (12%)
Self-pay	38 (6%)	16 (4%)	22 (8%)
Mental health diagnosis present (%)	217 (33%)	136 (36%)	81 (29%)
Prior period of abstinence present (%)	412 (62%)	248 (65%)	164 (58%)
Stage of change^1^ (%)			
Precontemplation	14 (2%)	9 (2%)	5 (2%)
Contemplation	96 (14%)	57 (15%)	39 (14%)
Preparation	367 (55%)	193 (50%)	174 (62%)
Action	122 (18%)	90 (24%)	32 (11%)
Maintenance	1 (<1%)	0 (0%)	1 (<1%)
Relapse	28 (4%)	16 (4%)	12 (4%)
Unknown	38 (6%)	3 (<1%)	20 (7%)
AUDIT-C, mean (SD)	6.4 (5.5)	6.8 (5.4)	5.8 (5.4)
Alcohol use disorder^1^ (%)	248 (37%)	167 (44%)	81 (29%)
Opioid use^2^(%)	291 (44%)	157 (41%)	134 (47%)
Cocaine use^2^(%)	174 (26%)	100 (26%)	74 (26%)
Cannabis use^3^ (%)	111 (17%)	64 (17%)	47 (17%)

*Note.* Some percentages may not equal 100% due to rounding error

1 Documented alcohol use disorder diagnosis

2 Past 12-month use or documented use disorder

3 No documented cannabis use disorder in the sample

Fifty-two percent of females (*n=*111) and 61% of males (*n*=278) met the criteria for at-risk drinking based on the AUDIT-C (≥3 for females; ≥4 for males). Females had a mean AUDIT-C score of 5.6 (SD=5.5; possible AUDIT-C range: 0-12) while the mean for males was 6.8 (SD=5.4). An alcohol use disorder diagnosis was documented in the problem list for 37% of the sample. Forty-one percent of the sample reported past 12-month opioid use (*n*=276), 26% (*n*=172) had past 12-month cocaine use, and 17% (*n*=111) reported past 12-month cannabis use. Use disorders other than alcohol were infrequently documented (e.g., opioid use disorder 5%; cocaine and cannabis both <1%).

### 3.2 Factors associated with being linked to treatment

Table 3 provides results from the multivariable logistic regression model examining factors associated with successful linkage to treatment. Patients with Medicaid had higher odds of being linked to treatment when compared to other insurance types (OR=2.94, 95% CI:2.09-4.13, p=<.001; among linked patients, 61% were Medicaid insured vs 33% among those not linked). The majority of those without Medicaid had employer-based or private insurance. Among those linked 25% had private insurance, relative to 47% among those not linked. Those with a documented alcohol use disorder diagnosis in the problem list had higher odds of being linked to treatment than those who did not (OR=1.59, 95% CI:1.07-2.35, p=.02; 44% of those linked had an alcohol use disorder diagnosis in the problem list relative to 29% among unlinked). When compared to all other stages of change, patients in the “action” stage had greater odds of being linked to treatment (OR=2.33, 95% CI:1.47-3.69, p=<.001). However, prevalence of the “action” stage was relatively low overall (24% in action stage among those liked vs 11% among those unlinked) because a small majority of patients (55% overall) were in the “preparation” stage of change.

**Table 3:** Logistic regression with outcome of successful linkage to treatment

Variable	Odds ratio	95% CI	*p*
Age	1.00	0.98-1.01	0.70
Male^1^	0.85	0.59-1.22	0.37
Race^2^			
Black	1.19	0.82-1.71	0.36
Other	1.47	0.71-3.07	0.30
Medicaid^3^	**2.94**	**2.09-4.13**	**<0.001**
Alcohol use disorder diagnosis^4^	**1.59**	**1.07-2.35**	**0.02**
Opioid use^4^	0.96	0.66-1.40	0.83
Cocaine use^4^	1.07	0.73-1.59	0.73
Cannabis use^4^	1.01	0.64-1.59	0.97
Mental health diagnosis^4^	1.25	0.93-1.68	0.13
Prior period of abstinence^4^	1.16	0.88-1.52	0.29
Action stage of change^5^	**2.33**	**1.47-3.69**	**<0.001**

*Note.***Bold** indicates significant at α = .05

1 Relative to female

2 Relative to White

3 Relative to other insurance

4 Relative to the absence of this factor

5 Relative to all other stages and NA/Not recorded

## 4 Discussion

This study examined factors associated with linkage to substance use treatment following brief intervention in the ED. We identified Medicaid insurance, having a documented alcohol use disorder diagnosis, and being in the “action” stage of change as factors associated with treatment linkage. These results provide preliminary information to optimize success of brief intervention and referral to treatment.

Prior data have demonstrated that patients with insurance are more likely to attend treatment following referral relative to uninsured patients (Blow et al., 2010). We expanded upon this knowledge by examining the role of insurance type. The association of Medicaid insurance with successful treatment linkage is novel with respect to ED settings but is consistent with prior data examining the role of Medicaid in substance use treatment. Data collected among a diverse sample of over 13,000 individuals in California found substance use programs that accept Medicaid tend to have shorter wait periods for intake appointments relative to programs that did not accept Medicaid (Guerrero, 2013), which may facilitate successful linkage to treatment. Maryland is a Medicaid-expansion state, and the authors’ clinical experience is that there are many treatment programs in the greater Baltimore area that accept Medicaid and fewer that accept Medicare. This geographic component may explain why higher rates of linkage to treatment were favorable for Medicaid public insurance in the present sample. Because persons with Medicaid have more ED visits and fewer office visits than those with private insurance (Allen et al., 2021), the prevalence of Medicaid insurance among the sample may reflect Medicaid patients’ greater reliance on the ED for care and referral whereas privately insured persons may have access to referral from other sources of care and thus be less inclined to accept an ED referral. Whether the private insurance patients in this sample are representative of those with private insurance more generally remains to be determined. Irrespective of the reason for discrepant linkage outcomes, it is important for clinicians to know that Medicaid patients may be more likely to benefit from this type of referral program. At the national level, Medicaid expansion has been associated with improved health outcomes and healthcare access (Cawley et al., 2018; Meinhofer & Witman, 2018; Sharp et al., 2018; Snider et al., 2019; Wen et al., 2020) which may have the potential to reduce substance-related ED visits. Overall, the present findings, along with prior literature comparing insured and uninsured patients, emphasizes the potential importance of insurance status for substance use patients and suggests encouraging uninsured ED patients to determine Medicaid eligibility.

Persons with a documented alcohol use disorder diagnosis in the problem list were also more likely to be linked to treatment. Alcohol use disorder tends to be under-detected in a clinical setting and under-documented in the health record relative to self-reported problem drinking (Mitchell et al., 2012). Thus, a documented alcohol use disorder diagnosis may reflect relatively greater clinical severity of presentation relative to self-reported assessments. Notably, this difference in severity between linked and unlinked patients was not detected by the AUDIT-C, which does not assess alcohol withdrawal. Severe alcohol withdrawal symptoms could be a driver of successful linkage to treatment if these were the reason for the ED visit. Thus, the present results may also be consistent with prior data showing that persons who needed more intensive intervention also showed greater reductions in alcohol use following brief intervention in the ED (Merchant et al., 2017). Although we do not know whether withdrawal or severity were underlying the relationship between treatment linkage and a documented alcohol use disorder diagnosis in our sample, formal assessment of alcohol use disorder severity and withdrawal may be considered in future analyses of linkage to treatment from the ED.

While controlling for an alcohol use disorder diagnosis and other variables, self-reported past 12-month cocaine, opioid, and cannabis use were not associated with treatment linkage. One possible reason for this is that a broad measure assessing past 12-month use may not be sufficiently sensitive to problematic use as a brief measure assessing frequency of substance use or functional impairment due to substance use. Further, it is important to note that there were low rates of clinical documentation for substance use disorders according to both patient self-report as well as absence from the general medical problem list. Although speculative, the lack of documentation for self-reported substance use may be related to the fast-paced environment and the prioritization of documentation of information that was more directly relevant to the brief intervention, rapport-building, and other medical care (Boyd et al., 2022; Simon et al., 2020). A substance use disorder may be especially underreported in electronic health records despite its clinical significance due to the time needed to assess substance use disorder and substance use disorder severity. There are other major systemic barriers for individuals reporting their substance use such as stigma, medical neglect, treatment that may be punitive (e.g., forced tapering), involvement with the criminal justice system, involvement with child protective services (especially for pregnant women), and impact on insurance rates/coverage (Boyd et al., 2022, McNeil et al., 2014; McNeil et al., 2016; Simon et al., 2020; van Olphen et al., 2006; van Olphen et al., 2009). In the future, it may be useful to determine methods to encourage patient disclosure and to remediate staff documentation, including strategies to reduce staff burden, in order to optimize care and determine whether either drug type or use severity is associated with linkage to treatment (Curtis et al., 2019; Duber et al., 2018; Melnick et al., 2022; Uong et al., 2022; Wamsley et al., 2018).

Although we included sex and race as factors in the analyses, we did not observe statistically significant differences in linkage to treatment as a function of these variables. As previously noted, sex and racial disparities in treatment receipt and referral have been documented in the emergency department referral to treatment setting and in national databases (Amaducci et al., 2020; Martin et al., 2021; Webb et al., 2021). The lack of association in the present study may be due to our smaller sample size relative and differences in sampling design (e.g., clinical data extracted from a single ED located in Maryland vs. nationally representative sampling frames) (Amaducci et al., 2020; Martin et al., 2021). Prior data observing racial disparities among Black patients referred to treatment by peer specialists was similar to the present study in terms of overall sample size, but the present study had a somewhat higher percentage of Black patients (32% vs. 21%) and the characteristics of the sample (e.g., geographic region) or intervention strategy may differ in ways that are not captured by the present analysis (Webb et al., 2021). We are not able to examine outcomes as a function of individual peer specialist or specialist-patient concordance with respect to racial and gender identity, however, we note that the peer specialist staff at JHBMC was diverse with respect to race and gender. Future interventions should evaluate the possible importance of patient-provider concordance on substance use treatment linkage outcomes (Otte, 2022).

The present data also provide further support for the “stages of change” model as a component of brief intervention and referral to treatment (Blow et al., 2010; Prochaska & DiClemente, 1983). Patients who were in the “action” stage were more likely to be linked to treatment relative to all other stages of change. Self-report of intention to take action including treatment entry are an important source of information in assessing prognosis for successful linkage. It is noteworthy that most patients were in the “preparation” stage of change, which is characterized by planning to change but not yet taking definitive action. Best practices may be to include a stages-of-change-guided intervention with a focus on intervention that can shift participants from preparation to the “action” stage of change. Recent data evaluating a 30-day stage-based text-message intervention showed feasibility and acceptability among patients who received brief intervention in primary care (Acquavita et al., 2021). The present data suggest that the ED may be another useful setting for stage-based interventions. The specific effects of peer recovery coaches or other “lived experience” models on patient stage of change have not been evaluated, but the present data suggest that stage of change is a potential therapeutic target that should be evaluated in the context of peer interventions. Other data evaluating interventions to improve linkage to treatment following brief intervention in the ED show follow-up with phone calls or text messages increase linkage to treatment (Kmiec & Suffoletto, 2019). Technology-based interventions may decrease implementation barriers related to provider time, training, and resources that may be current obstacles to widespread adoption of more intensive brief interventions with or without peer support (Acquavita et al., 2021).

The present study is limited by its reliance on retrospective rather than prospective data, and some variables were based on self-report and/or clinical judgment. Health record data are often incomplete and subject to underreporting due to high caseload or other factors. Not including a variable to capture the primary reason for treatment entry is another limitation that may be addressed in a future study. An individual entering treatment with a primary complaint of an arm injury may be in a different contemplative stage of change regarding substance use than someone entering with a primary complaint of alcohol use. Another limitation is the exclusion of patients who were referred to treatment but had missing or inconsistent values for treatment linkage, though our data analyses to evaluate the randomness of missing data did not identify any systematic differences in patient characteristics between included and excluded patients. Further, we were unable to evaluate the role of multiple ED visits or determine outcomes after initial linkage to treatment, such as treatment modality or engagement or decreased substance use. We were unable to evaluate differences between peer recovery coach led interventions relative to interventions initiated by other professionals, or to compare differences between specific peer specialists. Such comparisons would benefit from prospective randomized trials.

Study limitations are offset by the large sample of patients with confirmed treatment attendance or confirmed failure to attend. We were able to evaluate the relative predictive importance of multiple clinically relevant factors relevant to treatment attendance by including them in a single analysis. Data collected in a highly controlled research context may be ideal for determining mechanisms of change, but analyses of electronic health records such as the present study nevertheless provide data about what associations are knowable and informative in a real-world clinical data of a busy urban ED environment. Although imperfect, electronic health records account for the vast majority of data accessible to clinicians and are the backbone upon which predictive machine learning algorithms are currently being built to improve clinical care (Barenholtz et al., 2020; Ouchi et al., 2018). Thus, electronic health record data and controlled, prospective research data are both valuable to the empirical literature.

## 5 Conclusion

This study identified persons who were insured under Medicaid, those who had a documented alcohol use disorder diagnosis, and those who were in the “action” stage of change as more likely to be successfully linked to treatment following brief intervention in the ED. If prospective research studies confirm these associations, they could be used to inform ED protocols to reliably deliver standard interventions to patients with a high likelihood of attending treatment and provide more intensive supports to patients who are less likely to be linked to care.

## CRediT authorship contribution statement

**Orrin D. Ware:** Conceptualization, Methodology, Validation, Formal analysis, Data curation, Writing – original draft, Writing – review & editing, Visualization. **Megan E. Buresh:** Conceptualization, Methodology, Validation, Investigation, Data curation, Writing – review & editing, Funding acquisition. **Nathan A. Irvin:** Conceptualization, Methodology, Validation, Investigation, Data curation, Writing – review & editing, Funding acquisition. **Maxine L. Stitzer:** Conceptualization, Methodology, Resources, Writing – review & editing, Supervision, Funding acquisition. **Mary M. Sweeney:** Conceptualization, Methodology, Validation, Investigation, Resources, Data curation, Writing – original draft, Writing – review & editing, Visualization, Supervision, Project administration, Funding acquisition.

## Declaration of Competing Interest

No conflict declared.

## Appendix Supplementary materials

Image, application 1

## References

[bib0001] Acquavita S.P., Richardson G.B., Smith R., Van Loon R.A., Brehm B., Kim K., Diers T. (2021). Outcomes of an interprofessional SBIRT training program: Knowledge attainment and perceived competence for practice. Substance abuse.

[bib0002] Allen H., Gordon S.H., Lee D., Bhanja A., Sommers B.D. (2021). Comparison of Utilization, Costs, and Quality of Medicaid vs Subsidized Private Health Insurance for Low-Income Adults. JAMA Netw Open.

[bib0003] Amaducci A.M., Greenberg M.R., Sheen A.W., Warren H.R., Parikh P.M., Roth P., Weaver K.R., Richardson D.M., Burmeister D.B., Stephens J.L., Cannon R.D. (2020). Sex-specific outcomes in a substance use intervention program. Clin. Ther..

[bib0004] Bahorik A.L., Satre D.D., Kline-Simon A.H., Weisner C.M., Young-Wolff K.C., Campbell C.I. (2018). Alcohol, marijuana, and opioid use disorders: 5-Year patterns and characteristics of emergency department encounters. Substance abuse.

[bib0005] Barata I.A., Shandro J.R., Montgomery M., Polansky R., Sachs C.J., Duber H.C., Weaver L.M., Heins A., Owen H.S., Josephson E.B., Macias-Konstantopoulos W. (2017). Effectiveness of sbirt for alcohol use disorders in the emergency department: a systematic review. The western j. emerg. med..

[bib0006] Barbosa C., McKnight-Eily L.R., Grosse S.D., Bray J. (2020). Alcohol screening and brief intervention in emergency departments: review of the impact on healthcare costs and utilization. J. Subst. Abuse Treat..

[bib0007] Barenholtz E., Fitzgerald N.D., Hahn W.E. (2020). Machine-learning approaches to substance-abuse research: emerging trends and their implications. Curr. Opin. Psychiatry.

[bib0008] Blow F.C., Walton M.A., Murray R., Cunningham R.M., Chermack S.T., Barry K.L., Ilgen M.A., Booth B.M. (2010). Intervention attendance among emergency department patients with alcohol- and drug-use disorders. J*. stud. alcohol and drugs*.

[bib0009] Bogenschutz M.P., Donovan D.M., Mandler R.N., Perl H.I., Forcehimes A.A., Crandall C., Lindblad R., Oden N.L., Sharma G., Metsch L., Lyons M.S., McCormack R., Macias-Konstantopoulos W., Douaihy A. (2014). Brief intervention for patients with problematic drug use presenting in emergency departments: a randomized clinical trial. JAMA Intern Med.

[bib0010] Boyd J., Maher L., Austin T., Lavalley J., Kerr T., McNeil R. (2022). Mothers who use drugs: closing the gaps in harm reduction response amidst the dual epidemics of overdose and violence in a canadian urban setting. Am. J. Public Health.

[bib0011] Bradley K.A., DeBenedetti A.F., Volk R.J., Williams E.C., Frank D., Kivlahan D.R. (2007). AUDIT-C as a brief screen for alcohol misuse in primary care. Alcohol.: Clin. Exp. Res..

[bib0012] Bush K., Kivlahan D.R., McDonell M.B., Fihn S.D., Bradley K.A. (1998). The AUDIT alcohol consumption questions (AUDIT-C): an effective brief screening test for problem drinking. Ambulatory Care Quality Improvement Project (ACQUIP). Alcohol Use Disorders Identification Test. Arch. Intern. Med..

[bib0013] Cawley J., Soni A., Simon K. (2018). Third year of survey data shows continuing benefits of medicaid expansions for low-income childless adults in the U.S. J. Gen. Intern. Med..

[bib0014] Cowell A.J., Dowd W.N., Mills M.J., Hinde J.M., Bray J.W. (2017). Sustaining SBIRT in the wild: simulating revenues and costs for screening, brief intervention and referral to treatment programs. Addiction.

[bib0015] Curtis A.C., Satre D.D., Ly K., Wamsley M., Satterfield J. (2019). Implementation of alcohol and drug screening, brief intervention, and referral to treatment: nurse practitioner learner perspectives on a mobile app. J. Am. Assoc. Nurse Pract..

[bib0016] D'Onofrio G., Chawarski M.C., O'Connor P.G., Pantalon M.V., Busch S.H., Owens P.H., Hawk K., Bernstein S.L., Fiellin D.A. (2017). Emergency department-initiated buprenorphine for opioid dependence with continuation in primary care: outcomes during and after intervention. J. Gen. Intern. Med..

[bib0017] D'Onofrio G., O'Connor P.G., Pantalon M.V., Chawarski M.C., Busch S.H., Owens P.H., Bernstein S.L., Fiellin D.A. (2015). Emergency department-initiated buprenorphine/naloxone treatment for opioid dependence: a randomized clinical trial. JAMA.

[bib0018] Duber H.C., Barata I.A., Cioè-Peña E., Liang S.Y., Ketcham E., Macias-Konstantopoulos W., Ryan S.A., Stavros M., Whiteside L.K. (2018). Identification, management, and transition of care for patients with opioid use disorder in the emergency department. Ann. Emerg. Med..

[bib0019] Eddie D., Hoffman L., Vilsaint C., Abry A., Bergman B., Hoeppner B., Weinstein C., Kelly J.F. (2019). Lived experience in new models of care for substance use disorder: a systematic review of peer recovery support services and recovery coaching. Front. Psychol..

[bib0020] Guerrero E.G. (2013). Enhancing access and retention in substance abuse treatment: the role of Medicaid payment acceptance and cultural competence. Drug Alcohol Depend..

[bib0021] Hatch-Maillette M.A., Donovan D.M., Laschober T.C. (2020). Dosage of booster phone calls following an SBIRT intervention in the emergency department for reducing substance use. J. Subst. Abuse Treat..

[bib0022] Hawk K., D'Onofrio G (2018). Emergency department screening and interventions for substance use disorders. Addiction sci. clin. practice.

[bib0023] Holland K.M., Jones C., Vivolo-Kantor A.M., Idaikkadar N., Zwald M., Hoots B., Yard E., D'Inverno A., Swedo E., Chen M.S., Petrosky E., Board A., Martinez P., Stone D.M., Law R., Coletta M.A., Adjemian J., Thomas C., Puddy R.W., Peacock G., …, Houry D. (2021). Trends in US emergency department visits for mental health, overdose, and violence outcomes before and during the covid-19 pandemic. JAMA Psychiatry.

[bib0024] Kaczorowski J., Bilodeau J., M Orkin A., Dong K., Daoust R., Kestler A (2020). Emergency department-initiated interventions for patients with opioid use disorder: a systematic review. Acad. Emerg. Med..

[bib0025] Kaner E.F., Beyer F.R., Muirhead C., Campbell F., Pienaar E.D., Bertholet N., Daeppen J.B., Saunders J.B., Burnand B. (2018). Effectiveness of brief alcohol interventions in primary care populations. Cochrane. Database. Syst. Rev..

[bib0026] Karaca Z., Moore B.J. (2020). Costs of emergency department visits for mental and substance use disorders in the United States, 2017: Statistical Brief #257. Healthcare Cost and Utilization Project (HCUP) Statistical Briefs.

[bib0027] Kleinman M.B., Doran K., Felton J.W., Satinsky E.N., Dean D., Bradley V., Magidson J.F. (2021). Implementing a peer recovery coach model to reach low-income, minority individuals not engaged in substance use treatment. Substance abuse.

[bib0028] Kmiec J., Suffoletto B. (2019). Implementations of a text-message intervention to increase linkage from the emergency department to outpatient treatment for substance use disorders. J. Subst. Abuse Treat..

[bib0029] Landy M.S., Davey C.J., Quintero D., Pecora A., McShane K.E. (2016). A systematic review on the effectiveness of brief interventions for alcohol misuse among adults in emergency departments. J. Subst. Abuse Treat..

[bib0030] Liu S., Scholl L., Hoots B., Seth P. (2020). Nonfatal drug and polydrug overdoses treated in emergency departments - 29 States, 2018-2019. MMWR Morb. Mortal Wkly. Rep..

[bib0031] Martin C.E., Parlier-Ahmad A.B., Beck L., Scialli A., Terplan M. (2021). Need for and receipt of substance use disorder treatment among adults, by gender, in the United States. Public health reports (Washington, D.C. : 1974).

[bib0032] McCall M.H., Wester K.L., Bray J.W., Hanchate A.D., Veach L.J., Smart B.D., Wachter Morris C. (2021). SBIRT administered by mental health counselors for hospitalized adults with substance misuse or disordered use: Evaluating hospital utilization and costs. J. Subst. Abuse Treat..

[bib0033] McNeil R., Kerr T., Pauly B., Wood E., Small W. (2016). Advancing patient-centered care for structurally vulnerable drug-using populations: a qualitative study of the perspectives of people who use drugs regarding the potential integration of harm reduction interventions into hospitals. Addiction.

[bib0034] McNeil R., Small W., Wood E., Kerr T. (2014). Hospitals as a ‘risk environment’: an ethno-epidemiological study of voluntary and involuntary discharge from hospital against medical advice among people who inject drugs. Soc. Sci. Med..

[bib0035] Meinhofer A., Witman A.E. (2018). The role of health insurance on treatment for opioid use disorders: evidence from the affordable care act medicaid expansion. J. Health Econ..

[bib0036] Melnick E.R., Nath B., Dziura J.D., Casey M.F., Jeffery M.M., Paek H., Soares W.E., Hoppe J.A., Rajeevan H., Li F., Skains R.M., Walter L.A., Patel M.D., Chari S.V., Platts-Mills T.F., Hess E.P., D'Onofrio G. (2022). User centered clinical decision support to implement initiation of buprenorphine for opioid use disorder in the emergency department: EMBED pragmatic cluster randomized controlled trial. BMJ.

[bib0037] Merchant R.C., Romanoff J., Zhang Z., Liu T., Baird J.R. (2017). Impact of a brief intervention on reducing alcohol use and increasing alcohol treatment services utilization among alcohol- and drug-using adult emergency department patients. Alcohol.

[bib0038] Merchant R.C., Zhang Z., Zhang Z., Liu T., Baird J.R. (2018). Lack of efficacy in a randomised trial of a brief intervention to reduce drug use and increase drug treatment services utilisation among adult emergency department patients over a 12-month period. Emerg Med J.

[bib0039] Monico L.B., Oros M., Smith S., Mitchell S.G., Gryczynski J., Schwartz R. (2020). One million screened: Scaling up SBIRT and buprenorphine treatment in hospital emergency departments across Maryland. Am. J. Emerg. Med..

[bib0040] Moore B.J., Stocks C., Owens P.L. (2017). https://www.hcup-us.ahrq.gov/reports/statbriefs/sb227-Emergency-Department-Visit-Trends.pdf.

[bib0041] Otte S.V. (2022). Improved patient experience and outcomes: is patient-provider concordance the key?. J. Patient Exp..

[bib0042] Ouchi K., Lindvall C., Chai P.R., Boyer E.W. (2018). Machine learning to predict, detect, and intervene older adults vulnerable for adverse drug events in the emergency department. J. Med. Toxicol..

[bib0043] Pringle J.L., Kelley D.K., Kearney S.M., Aldridge A., Dowd W., Johnjulio W., Venkat A., Madden M., Lovelace J. (2018). Screening, brief intervention, and referral to treatment in the emergency department: an examination of health care utilization and costs. Med. Care.

[bib0044] Prochaska J.O., DiClemente C.C. (1983). Stages and processes of self-change of smoking: toward an integrative model of change. J. Consult. Clin. Psychol..

[bib0045] Saitz R. (2018). Absence of a quick fix does not mean 'do nothing:' time to address drug use in the ED. Emerg. Med. J..

[bib0046] Sanjuan P.M., Rice S.L., Witkiewitz K., Mandler R.N., Crandall C., Bogenschutz M.P. (2014). Alcohol, tobacco, and drug use among emergency department patients. Drug Alcohol Depend..

[bib0047] Sharp A., Jones A., Sherwood J., Kutsa O., Honermann B., Millett G. (2018). Impact of medicaid expansion on access to opioid analgesic medications and medication-assisted treatment. Am. J. Public Health.

[bib0048] Siegel R., Sullivan N., Monte A.A., Vargas N.M., Cooper Z.D., Ma Y., Meltzer A.C. (2021). Motivational interviewing to treat substance use disorders in the emergency department: a scoping review. Am. J. Emerg. Med..

[bib0049] Simon R., Snow R., Wakeman S. (2020). Understanding why patients with substance use disorders leave the hospital against medical advice: a qualitative study. Substance abuse.

[bib0050] Snider J.T., Duncan M.E., Gore M.R., Seabury S., Silverstein A.R., Tebeka M.G., Goldman D.P. (2019). Association between state medicaid eligibility thresholds and deaths due to substance use disorders. JAMA Netw. Open.

[bib0051] Soares W.E., Melnick E.R., Nath B., D'Onofrio G., Paek H., Skains R.M., Walter L.A., Casey M.F., Napoli A., Hoppe J.A., Jeffery M.M. (2022). Emergency department visits for nonfatal opioid overdose during the covid-19 pandemic across six us health care systems. Ann. Emerg. Med..

[bib0052] Substance Abuse and Mental Health Services Administration (2022). https://www.samhsa.gov/sbirt.

[bib0053] Uong S., Tomedi L.E., Gloppen K.M., Stahre M., Hindman P., Goodson V.N., Crandall C., Sklar D., Brewer R.D. (2022). Screening for excessive alcohol consumption in emergency departments: a nationwide assessment of emergency department physicians. J. Public Health Manag. Pract..

[bib0054] van Olphen J., Freudenberg N., Fortin P., Galea S. (2006). Community reentry: perceptions of people with substance use problems returning home from New York City jails. J. Urban Health.

[bib0055] van Olphen J., Eliason M.J., Freudenberg N., Barnes M. (2009). Nowhere to go: how stigma limits the options of female drug users after release from jail. Subst. Abuse Treat. Prev. Pol..

[bib0056] Vivolo-Kantor A.M., Hoots B.E., Scholl L., Pickens C., Roehler D.R., Board A., Mustaquim D., Smith H., Snodgrass S., Liu S. (2020). Nonfatal drug overdoses treated in emergency departments - United States, 2016-2017. MMWR. Morbidity and mortality weekly report.

[bib0057] Vivolo-Kantor A.M., Seth P., Gladden R.M., Mattson C.L., Baldwin G.T., Kite-Powell A., Coletta M.A. (2018). Vital signs: trends in emergency department visits for suspected opioid overdoses - United States, July 2016-September 2017. MMWR. Morbidity and mortality weekly report.

[bib0058] Waller R., Bonar E.E., Fernandez A.C., Walton M.A., Chermack S.T., Cunningham R.M., Blow F.C. (2019). Exploring the components of an efficacious computer brief intervention for reducing marijuana use among adults in the emergency department. J. Subst. Abuse Treat..

[bib0059] Wamsley M., Satterfield J.M., Curtis A., Lundgren L., Satre D.D. (2018). Alcohol and drug screening, brief intervention, and referral to treatment (sbirt) training and implementation: perspectives from 4 Health Professions. J Addict Med.

[bib0060] Webb C.P., Huecker M., Shreffler J., McKinley B.S., Khan A.M., Shaw I. (2021). Racial disparities in linkage to care among patients with substance use disorders. J. Subst. Abuse Treat..

[bib0061] Wen H., Soni A., Hollingsworth A., Freedman S., Benitez J., Simon K., Saloner B. (2020). Association Between Medicaid Expansion and Rates of Opioid-Related Hospital Use. JAMA Intern Med.

[bib0062] White A.M., Slater M.E., Ng G., Hingson R., Breslow R. (2018). Trends in alcohol-related emergency department visits in the united states: results from the nationwide emergency department sample, 2006 to 2014. Alcohol Clin. Exp. Res..

